# Decision Fatigue Among Chinese Nurse Managers: A Multicenter Cross‐Sectional Study on Implicit Cognitive Load Determinants

**DOI:** 10.1155/jonm/6980432

**Published:** 2026-08-23

**Authors:** Wenyu Li, Menfang Li, Jingjing Ma, Xiaoyu Zhou, Yani Yan, Shaozhi Tian, Kemiao Dai, Zhiyang Xie, Huirong Xie, Chunqing Yang, Longcha Liu, Zhengjie Dai, Sula Li, Xiaoqun Xu

**Affiliations:** ^1^ The First Affiliated Hospital of Wenzhou Medical University, Wenzhou, China, wzhospital.cn; ^2^ Ningbo Medical Center LiHuili Hospital, Ningbo, China, nbws.gov.cn; ^3^ The First Affiliated Hospital of Zhejiang University, Hangzhou, China, zju.edu.cn; ^4^ Wenzhou Integrated Traditional Chinese and Western Medicine Hospital, Wenzhou, China; ^5^ Lishui Central Hospital, Lishui, China, lshospital.zj.cn

**Keywords:** decision fatigue, head nurses, nurse management, practice environment, psychological training

## Abstract

**Background:**

Decision fatigue, conceptualized within Baumeister’s strength model of self‐control, describes the deterioration of decision‐making quality following protracted choice‐making processes. The intensive cognitive and managerial demands inherent in nursing leadership render head nurses particularly susceptible to this phenomenon, with subsequent deleterious effects on patient safety, staff well‐being, and organizational performance.

**Aims:**

This study aims to delineate the characteristics of decision fatigue among head nurses and identify the specific occupational and environmental factors associated with this phenomenon, particularly within the intricacies of complex clinical management. Furthermore, the research seeks to provide a robust evidence base for healthcare administrators to inform the design and implementation of targeted intervention strategies aimed at mitigating cognitive exhaustion in nurse leaders.

**Methods:**

A descriptive, multicenter, cross‐sectional study was conducted. Data were collected between November and December 2024 across 17 healthcare institutions in China. A total of 144 head nurses were recruited via convenience sampling. Data were collected using a sociodemographic questionnaire, the Decision Fatigue Scale (DFS), and the Nurse Manager Practice Environment Scale (NMPES). Statistical analyses included Mann–Whitney U and Kruskal–Wallis H tests for group comparisons, Spearman correlation, and multiple linear regression to identify independent predictors.

**Results:**

Participants reported low‐to‐moderate levels of decision fatigue, with a median score of 9 (IQR = 7.25–10.75), while practice environment scores were relatively optimal (median = 225, IQR = 216–249). Multiple linear regression identified cultural heritage (*β* = −0.296), physical status (absence of fatigue) (*β* = −0.234), and organizational planning ability (*β* = −0.228) as significantly associated with lower decision fatigue. Notably, head nurses in tertiary hospitals exhibited significantly higher fatigue levels (median = 10, IQR = 8.5–12.5), underscoring the impact of systemic institutional pressures.

**Conclusions:**

Decision fatigue may be understood through a proposed dual‐pathway framework: a direct pathway involving task overload and ego depletion and an indirect pathway involving emotional labor and cognitive interference. Psychological training was associated with lower fatigue levels, potentially by facilitating cognitive reappraisal of stress. Effective mitigation strategies may benefit from a multifaceted approach incorporating situational leadership and nonmonetary support. Furthermore, cultural heritage and organizational planning ability were identified as factors associated with reduced fatigue, while addressing structural constraints may help optimize decision‐making efficacy among nurse leaders.

**Implications for Nursing Management:**

The identified three‐tier protective framework—comprising cultural heritage, organizational planning ability, and physical resilience—offers an actionable framework for intervention. Supported by a robust cultural heritage, nurse managers may benefit from structured daily planning, algorithmic cognitive outsourcing, and proactive fatigue self‐monitoring. At the institutional level, administrators could consider formalizing mentorship programs, integrating evidence‐based psychological training, and addressing structural staffing constraints. Furthermore, policymakers might re‐examine resource allocation strategies that disproportionately burden midtier hospital leaders. Multilevel, coordinated interventions may be essential to safeguard head nurse decision‐making capacity, patient safety, and staff well‐being.

**Summary**
• What is already known
◦ Decision fatigue affects cognitive performance and decision‐making abilities in high‐stress professions.
◦ Nurse leaders frequently face significant decision‐making challenges that can lead to mental exhaustion, subsequently impacting their managerial efficacy*.*
◦ There remains a lack of comprehensive understanding regarding how specific practice environments and systemic conditions influence decision fatigue among frontline nurse leaders.
• What this paper adds
◦ This study identifies robust cultural heritage and individual organizational planning ability as key factors significantly associated with lower decision fatigue in nurse managers.
◦ The research proposes a theoretical dual‐pathway framework (direct task overload vs. indirect emotional labor) to better understand how decision fatigue may manifest in clinical leadership.
◦ The findings suggest that nonmonetary support, structured mentorship, and targeted psychological training may play crucial roles in mitigating decision fatigue within healthcare settings.

## 1. Background

Decision fatigue, conceptualized within Baumeister’s strength model of self‐control, describes the gradual deterioration in decision‐making quality and self‐regulatory capacity resulting from repeated acts of choice [1, 2]. This state manifests as a diminished capacity for deliberative reasoning, often leading to impulsive or avoidant decisions [3] and impairing critical cognitive functions such as working memory [4].

In healthcare, where clinical and managerial decisions directly impact patient safety and organizational outcomes [5], mitigating decision fatigue is paramount. Reflecting its significance, the U.S. National Institute of Nursing Research has prioritized decision science to advance nursing practice. While fatigue among bedside nurses is well documented, nurse leaders—who are equally susceptible—have received less empirical scrutiny [6]. Head nurses (internationally referred to as frontline nurse managers or unit managers) are pivotal in this leadership stratum, overseeing unit operations, resource allocation, and staff leadership [7]. Acting as the essential liaison between senior administration and clinical staff, they navigate complex challenges, including conflict mediation and diverse stakeholder needs [8], a role necessitating high levels of ethical and transformational leadership.

In China, the role of the “Hushizhang” (head nurse) is distinctly shaped by the institutional landscape of the public healthcare system. Characterized by systemic pressures and resource constraints, these managers must reconcile top‐down administrative mandates (e.g., rigid efficiency targets) with bottom‐up interpersonal demands (e.g., mediating doctor–nurse–patient dynamics) [8]. This role is embedded within a hierarchical framework and a collectivist culture, requiring a nuanced balance between authoritative leadership and interpersonal harmony.

Crucially, the intersection of professional demands and demographic stressors warrants investigation. The head nurse population is predominantly female. In addition to universal professional pressures, female leaders—particularly in the Chinese context—often face a “double burden” regarding traditional domestic and child‐rearing responsibilities [9]. This compounding stress may accelerate cognitive depletion. Therefore, including demographic variables such as gender and childcare status is essential to understanding the comprehensive mental load of this population.

Despite the exacting nature of their role, research addressing decision fatigue among head nurses remains scarce in China. This gap limits the development of targeted interventions. Therefore, this study aims to investigate decision fatigue levels among Chinese head nurses and identify key practice environment determinants to inform contextually tailored support strategies.

## 2. Aim

This study aims to characterize decision fatigue among head nurses and identify specific workplace conditions and environmental factors associated with this state. Furthermore, we intend to provide hospital administrators and policymakers with evidence‐based insights to inform the development of targeted intervention strategies. The ultimate goal is to mitigate decision fatigue and its associated health risks, thereby optimizing job satisfaction and overall well‐being among nursing leadership.

### 2.1. Research Hypotheses

#### 2.1.1. Research Objective (Prevalence)

To describe the prevalence, distribution, and severity of decision fatigue among Chinese head nurses as measured by the Decision Fatigue Scale (DFS).

#### 2.1.2. Hypothesis (Associated Factors)

 H0: Decision fatigue levels do not differ significantly across demographic, occupational, or organizational characteristics.
 H1: Decision fatigue levels vary significantly across distinct demographic and organizational subgroups (e.g., hospital type, leadership training history, and organizational planning ability).

## 3. Methods

### 3.1. Setting and Participants

This multicenter, cross‐sectional study recruited head nurses from 17 hospitals across China between November and December 2024 using convenience sampling. The recruitment process was facilitated through the research team’s professional network. Specifically, the principal investigator contacted the nursing directors or administrators of the participating institutions to obtain institutional support. Subsequently, the survey—including the study objectives, informed consent protocols, and an anonymous link—was disseminated to eligible candidates via two primary channels: (1) hospital‐authorized WeChat work groups or (2) official nursing department emails. Participation was entirely voluntary, and all respondents provided electronic informed consent prior to completing the questionnaire.

### 3.2. Inclusion and Exclusion Criteria

The inclusion criteria were (1) currently serving as a head nurse, (2) possessing a minimum of 1 year of experience in nursing management, and (3) proficiency in reading and completing the survey in Chinese. Exclusion criteria comprised (1) a history of significant physical or mental illness, (2) current or planned extended leave during the study period, and (3) roles outside of frontline clinical management, such as exclusive engagement in research or teaching.

Sample size was determined using a general mean estimation formula [10]: $n=\left(\mu_{\alpha /2}^{2}\sigma^{2}\right)/\delta^{2}$. With *α* set at 0.05 (*μ*_*α*/2_ = 1.96), a standard deviation *σ* of 3.08, and a permissible error *δ* of 0.6—derived from a pilot study of 30 head nurses in October 2024—the minimum required sample size was calculated as 101. Accounting for a 20% nonresponse or attrition rate, a target sample of 127 was established. Ultimately, 144 head nurses were enrolled in the study.

### 3.3. Data Collection Tools

Data were collected using a comprehensive sociodemographic and occupational profile, the DFS, and the Nurse Manager Practice Environment Scale (NMPES).

#### 3.3.1. Sociodemographic and Occupational Profile

This instrument captured participants’ personal and professional characteristics. Variables were clustered into four categories: (1) Demographic covariates: age, gender, education, marital status, and number of children were included [6, 11]. (2) Work‐related context: institution type, specialty, income, departmental consistency, and years of management experience. Notably, “psychology training” was assessed as a self‐reported dichotomous variable indicating whether participants had received psychological education beyond standard clinical nursing training (formal certifications, workshops, or self‐directed learning). (3) Health and well‐being indicators: health status, fatigue levels, exercise frequency, and sleep duration [12]. (4) Exploratory variables: blood type was included as an exploratory variable to verify potential links to stress coping styles in specific cultural contexts [13].

#### 3.3.2. The DFS

Developed by Hickman et al. [14], this unidimensional scale assesses decision fatigue. The validated Chinese version demonstrates strong psychometric properties, with a Cronbach’s alpha of 0.854 [15]. The DFS comprises nine items rated on a 4‐point Likert scale (0 = “*strongly disagree*” to 3 = “*strongly agree*”). Total scores range from 0 to 27. In the present study, the Cronbach’s alpha was 0.905.

#### 3.3.3. NMPES

Developed by Warshawsky et al. [16] and culturally adapted by He and Hui [17], this scale comprises 45 items across eight dimensions. Responses are recorded on a 6‐point Likert scale. In this study, the Cronbach’s alpha was 0.961.

### 3.4. Data Collection Method

Electronic data collection was conducted via the Wenjuanxing platform. Eligible participants received a survey link and participated on a voluntary basis. The questionnaire was structured into three sections: (1) study objectives, data protection policies, and informed consent protocols; (2) instructions for scoring; and (3) the survey instruments. To ensure data integrity and prevent duplicate submissions, IP address restrictions were implemented, allowing only one submission per device.

### 3.5. Data Analysis

Data were analyzed using SPSS Version 25.0. Normality testing revealed that the continuous variables followed a nonnormal distribution; therefore, they are presented as medians and interquartile ranges (IQR). Categorical data are described using frequencies and percentages. Nonparametric tests, including the Mann–Whitney U test and Kruskal–Wallis H test, were employed for subgroup comparisons. The relationship between decision fatigue and the practice environment was evaluated via Spearman’s rank correlation analysis. Finally, multiple linear regression was performed to identify independent determinants of decision fatigue. Statistical significance was set at *p* < 0.05.

For the multiple linear regression analysis, all 16 variables that demonstrated statistically significant associations with decision fatigue in univariate analyses (Tables 1 and 2) and Spearman correlation analyses (Table 3) were entered into the initial model using the enter method (forced entry). This approach was chosen over stepwise selection to minimize the risk of capitalizing on chance variations and to preserve the theoretical integrity of the predictors identified in previous research. Prior to model interpretation, multicollinearity was assessed using tolerance and variance inflation factor (VIF) values, with a VIF > 10 considered indicative of significant multicollinearity. Model fit was evaluated using the coefficient of determination (*R* ^2^) and adjusted *R* ^2^, with the overall model significance tested via the F‐statistic. Residual diagnostics, including normality of residuals (Shapiro–Wilk test), homoscedasticity (Breusch–Pagan test), and independence of errors (Durbin–Watson statistic), were performed to validate the assumptions of linear regression.

**TABLE 1:** Comparison of TDF scores across institutional and professional characteristics (*N* = 144).

Item	*n* (%)	TDF scores	Statistic	*p*
Type of hospital				
Tertiary care hospital	100 (69.44)	9 (6.25–10)	*H* = 10.79	0.013
Regional tertiary hospital	25 (17.36)	10 (8.5–12.5)		
General hospital	13 (9.03)	10 (9–13)		
Community hospital	6 (4.17)	9 (5.25–9)		
Department				
Internal medicine	51 (35.42)	9 (7–11)	*H* = 5.502	0.703
Surgery	35 (24.31)	9 (8–10)		
Obstetrics and gynecology	9 (6.25)	9 (8–9.5)		
Pediatrics (including NICU)	6 (4.17)	9 (4.5–9.25)		
Emergency	8 (5.56)	10 (1.75–11.75)		
Outpatient (including infusion/preadmission center)	9 (6.25)	9 (7.5–9.5)		
Special departments (including endoscopy, supply room, catheterization, hemodialysis, reproductive center, etc.)	12 (8.33)	9 (7.5–11.5)		
Intensive care unit (ICU/EICU/CCU/RICU/Neuro ICU)	11 (7.64)	10 (9–15)		
Operating and recovery room	3 (2.08)	10 (9–11)^1^		
Monthly income (CNY)				
< 10,000	49 (34.03)	9 (8.5–10.5)	*H* = 2.302	0.316
10,000–20,000	85 (59.03)	9 (7–11)		
> 20,000	10 (6.94)	8 (4.75–10)		
Consistency with previous work experience				
Completely consistent	72 (50)	9 (8–10.75)	*H* = 2.132	0.344
Partially consistent	63 (43.75)	9 (7–10)		
Not consistent	9 (6.25)	9 (7.5–15.5)		
Specific management or leadership training				
Yes	124 (86.11)	9 (7–10)	*Z* = −1.96	0.05
No	20 (13.89)	9.5 (9–12.75)		
Psychology‐related training				
Yes	84 (58.33)	9 (7–10)	*Z* = −2.618	0.009
No	60 (41.67)	10 (9–11.75)		
Organizational and planning ability				
Very organized and planned	21 (14.58)	9 (2–9)	*H* = 25.03	< 0.001
Generally organized and planned	111 (77.08)	9 (8–10)		
Sometimes or rarely organized and planned	12 (8.33)	12 (11–14.75)		

^1^Due to the insufficient sample size in the operating and recovery room subgroup (*n* = 3), interquartile ranges could not be reliably estimated. Values for this subgroup are instead reported as median (minimum–maximum). Subgroup comparisons involving this category should be interpreted with caution, as findings remain preliminary pending validation in larger cohorts.

**TABLE 2:** Comparison of TDF scores across health and well‐being indicators (*N* = 144).

Item	*n* (%)	TDF scores	Statistics	*p*
Health status				
Very healthy	29 (20.13)	9 (5.5–10)	*Z* = −2.155	0.031
Some health issues	115 (79.86)	9 (8–11)		
Frequency of fatigue or other physical symptoms				
Never, always energetic	3 (2.08)	2 (0–5)	*H* = 15.94	0.003
Rarely, occasional fatigue	43 (29.86)	9 (7–10)		
Sometimes, several days a week	63 (43.75)	9 (9–11)		
Often, most of the time	31 (21.52)	10 (7–13)		
Always, severely impacts daily activities	4 (2.78)	10 (9–14)		
Frequency of weekly exercise				
1‐2 times	72 (50.00)	9 (8–11)	*H* = 5.45	0.142
2–4 times	30 (20.83)	9 (5–10)		
More than 4 times	3 (2.08)	7 (5–9)^1^		
No exercise	39 (27.08)	9 (7–11)		
Job satisfaction				
Very satisfied	14 (9.72)	9 (6.5–9.5)	*H* = 20.057	< 0.001
Satisfied	87 (60.41)	9 (6–10)		
Neutral or dissatisfied	43 (29.86)	11 (9–12)		
Work pressure				
No or little pressure	80 (55.56)	10 (9–14)	*H* = 12.699	0.002
Moderate pressure	41 (28.47)	9 (9–11)		
High pressure	23 (15.97)	10 (9–14)		
Current psychological status				
Very good	5 (3.47)	7 (1–9.5)	*H* = 10.889	0.004
Good	99 (68.75)	9 (7–10)		
Average or poor	40 (27.78)	10 (8.25–12.75)		
Nightly sleep duration				
4–6 h	46 (31.94)	9 (7–11)	*H* = 2.30	0.317
6–8 h	97 (67.36)	9 (8.5–10)		
> 8 h	1 (0.69)	2		

^1^Due to the small sample size in this subgroup (*n* = 3), quartiles could not be reliably estimated by SPSS. Values are reported as median (minimum–maximum) instead of median (IQR). Subgroup comparisons involving this category should be interpreted with caution.

**TABLE 3:** Spearman correlations between decision fatigue and practice environment dimensions (*N* = 144).

Item	Decision fatigue	
	*r*	*p*
Total practice environment	−0.409	< 0.001
Building a safety culture	−0.353	< 0.001
Relationship with nursing director	−0.300	< 0.001
Cultural heritage	−0.451	< 0.001
Sufficient human and material resources	−0.221	< 0.05
Organizational culture building	−0.405	< 0.001
Relationship with doctors	−0.322	< 0.001
Relationship with nurses	−0.323	< 0.001
Fair and manageable workload	−0.234	< 0.05

### 3.6. Ethical and Institutional Approvals

This study was conducted in strict accordance with the ethical regulations of the Chinese National Health Commission and the principles of the Declaration of Helsinki. The study protocol received formal approval from the Ethics Committee of Clinical Research (ECCR) of the First Affiliated Hospital of Wenzhou Medical University on June 6, 2024 (Approval No. 2024 (205)), serving as the Institutional Review Board (IRB) equivalent. Furthermore, the study was prospectively registered in the National Registry of Medical Research (PID: 305854). Rigorous safeguards (voluntariness, informed consent, anonymity, and independence of data collection) were implemented to protect participants.

## 4. Results

### 4.1. Decision Fatigue and Practice Environment Levels

The median total score for decision fatigue was 9 (IQR = 7.25–10.75), with a mean item score of 0.99 ± 0.14 (range: 0.84–1.24). Consistent with the scoring framework of the DFS [14], in which total scores range from 0 to 27, this median score reflects a low‐to‐moderate level of decision fatigue within the current sample.

The median total score for the practice environment was 225 (IQR = 216–249), with a median item score of 5.17 (IQR = 4.99–5.29). Given the NMPES theoretical range of 45–270, a median score of 225 places the perceived environment within the upper tertile of the scale. This indicates that the head nurses generally characterized their practice environment as highly favorable. Detailed scores for individual dimensions, as well as bivariate correlations between decision fatigue and the practice environment, are summarized in Tables 3, 4, and 5.

**TABLE 4:** Scores of Decision Fatigue Scale items (*N* = 144).

Items	No. items	Score range	Score	Mean item score	Avg. total score
Total decision fatigue	9	0–27	9 (7.25–10.75)	0.99 ± 0.14	128 (8.92)
Making decisions takes too much effort					179 (1.24)
I can’t decide which option is best					174 (1.21)
I can’t make decisions due to exhaustion and stress					146 (1.01)
My mood makes it difficult to decide					140 (0.97)
I lack confidence to make the right decision					137 (0.95)
Decisions are hard due to lack of focus					137 (0.95)
I make decisions without thorough consideration					129 (0.9)
Someone else should make decisions for me					122 (0.85)
I struggle to absorb and use information to decide					121 (0.84)

**TABLE 5:** Scores of the Nurse Manager Practice Environment Scale dimensions (*N* = 144).

Items	No. items	Score range	Score	Avg. item score
Total practice environment	45	148–270	225 (216–249)	5.17 (4.99–5.29)
Relationship with nurses	3	12–18	17 (15–18)	5.42 ± 0.17
Organizational culture building	4	16–24	22 (20–24)	5.42 ± 0.14
Cultural heritage	6	18–36	31 (29–34)	5.20 ± 0.20
Relationship with doctors	3	4–18	15 (15–18)	5.20 ± 0.09
Relationship with nursing director	6	14–36	35 (30–36)	5.16 ± 0.12
Building a safety culture	15	52–90	75 (71–82)	5.06 ± 0.39
Fair and manageable workload	4	11–24	20 (18–21)	4.84 ± 0.32
Sufficient human and material resources	4	5–24	17 (14–20)	4.30 ± 0.54

### 4.2. Demographic and Occupational Characteristics

Table 6 summarizes the general information of the participants. The study sample was predominantly female (95.14%), primarily aged 40–49 years (63.89%), and mostly held a bachelor’s degree or below (90.28%). Regarding family status, 55.56% of the participants were raising minor children. In terms of professional background, the largest proportion of participants had 21–30 years of total work experience (45.14%), while tenure as a head nurse was most commonly distributed between ≤ 5 years (34.72%) and 11–20 years (34.72%).

**TABLE 6:** Comparison of TDF scores across demographic and occupational characteristics (*N* = 144).

Items	*n* (%)	TDF scores	Statistic	*p*
Gender				
Male	7 (4.86)	9 (4–10)	*Z* = −0.326	0.745
Female	137 (95.14)	9 (7.5–11)		
Age (years)				
< 30	1 (0.69)	9	*H* = 4.292	0.232
30–39	20 (13.89)	9.5 (9–13.75)		
40–49	92 (63.89)	9 (8–10.75)		
50–59	31 (21.53)	9 (5–10)		
Education				
Bachelor or below	130 (90.28)	9 (7–10.25)	*Z* = −0.202	0.84
Master or above	14 (9.72)	9 (8.75–11)		
Children status				
Yes, adult	59 (40.97)	9 (7–10)	*Z* = −1.339	0.18
Yes, minor	84 (55.56)	9 (8–11)		
Number of children				
1 child	85 (59.03)	9 (7.5–10)	*Z* = −1.03	0.303
2 children	58 (40.28)	9 (7.75–11)		
Blood type				
Type O	59 (40.97)	9 (8–11)	*H* = 0.474	0.925
Type A	32 (22.22)	9 (5–11)		
Type B	38 (26.39)	9 (7.75–10.25)		
Type AB	15 (10.42)	9 (5–10)		
Years as head nurse				
≤ 5	50 (34.72)	9 (7–11.25)	*H* = 5.217	0.157
6–10	29 (20.14)	9 (9–11.50)		
11–20	50 (34.72)	9 (8.75–10)		
> 20	15 (10.42)	7 (3–10)		
Work experience (years)				
10–20	51 (35.42)	9 (7–11)	*H* = 5.036	0.081
21–30	65 (45.14)	9 (9–11)		
> 30	28 (19.44)	9 (5–10)		

Regarding decision fatigue, statistical analysis revealed no significant differences in total scores across gender, age, education level, childcare status, blood type, or occupational characteristics (all *p* > 0.05).

However, the impact of years of work experience on decision fatigue approached statistical significance (*H* = 5.036, *p* = 0.081). Participants with 21–30 years of experience reported nominally higher median scores compared to those with over 30 years of experience. This suggests potential heterogeneity in cognitive load among long‐term employees that warrants further investigation.

### 4.3. Impact of Institutional and Professional Characteristics on Decision Fatigue

Table 1 presents the comparison of total decision fatigue (TDF) scores across institutional types, clinical departments, and professional development backgrounds. The analysis revealed no statistically significant differences in TDF scores based on clinical department, average monthly income, or the consistency between current and prior work experience (all *p* > 0.05).

In contrast, several factors were identified as having statistically significant associations with decision fatigue (*p* < 0.05). Hospital type was significantly associated with TDF levels (*H* = 10.79, *p* = 0.013), with head nurses in regional tertiary and general hospitals reporting nominally higher fatigue scores compared to those in community settings. Professional development also played a critical role: participants who had received specific management or leadership training (*p* = 0.050) or psychology‐related training (*p* = 0.009) exhibited significantly lower fatigue levels.

Furthermore, self‐reported organizational and planning ability emerged as a highly significant factor (*H* = 25.03, *p* < 0.001). Head nurses who perceived themselves as “sometimes or rarely organized” reported the highest median TDF scores (12, IQR = 11–14.75), whereas those who identified as “very organized” maintained the lowest scores. These findings suggest that structured training and individual managerial competencies may serve as vital resources in mitigating decision fatigue.

### 4.4. Association Between Health‐Related Indicators and Decision Fatigue

Table 2 summarizes the variations in TDF scores based on participants’ health and lifestyle indicators. While weekly exercise frequency and nightly sleep duration showed no statistically significant associations with TDF (*p* > 0.05), several psychosomatic and professional factors emerged as significant predictors.

Notably, the subjective health status (*Z* = −2.155, *p* = 0.031) and the frequency of fatigue or physical symptoms (*H* = 15.94, *p* = 0.003) were significantly linked to decision fatigue levels. Participants who reported persistent fatigue exhibited the highest TDF scores (median = 10, IQR = 9–14). Furthermore, job satisfaction (*H* = 20.057, *p* < 0.001) and perceived work pressure (*H* = 12.699, *p* = 0.002) demonstrated strong associations with fatigue levels. Specifically, those who felt neutral or dissatisfied with their roles reported significantly higher TDF scores (median = 11) than their satisfied counterparts.

Finally, current psychological status was found to be a highly significant factor (*H* = 10.889, *p* = 0.004), with those reporting average or poor psychological well‐being experiencing greater decision‐making strain. These results suggest that decision fatigue among head nurses is closely intertwined with their overall mental health and professional fulfillment.

### 4.5. Multiple Linear Regression Analysis of Factors Associated With Decision Fatigue

#### 4.5.1. Variable Selection and Model Entry

Table 7 summarizes the 16 independent variables entered into the initial multiple linear regression model. These variables were selected based on their statistically significant associations with decision fatigue identified during univariate comparisons (Tables 1 and 2) and Spearman correlation analysis (Table 3).

**TABLE 7:** Independent variables included in the multiple linear regression model.

Variable	Category	Source (table)	*p*
Practice Environment and Job Factors			
Hospital type	Practice environment	5	0.013
Specific management/leadership training	Training	5	0.05
Psychology‐related training	Training	5	0.009
Organizational and planning ability	Skill	5	< 0.001
Cultural heritage	Practice environment	3	< 0.001
Sufficient human and material resources	Practice environment	3	< 0.05
Organizational culture building	Practice environment	3	< 0.001
Relationship with doctors	Practice environment	3	< 0.001
Relationship with nurses	Practice environment	3	< 0.001
Fair and manageable workload	Practice environment	3	< 0.05
Health and Psychological Status			
Current health status	Health	6	0.031
Frequency of fatigue or physical symptoms	Health	6	0.003
Current job satisfaction	Psychological	6	< 0.001
Current work stress	Psychological	6	0.002
Current psychological status	Psychological	6	0.004
Safety Culture and Leadership			
Building a safety culture	Practice environment	3	< 0.001
Relationship with nursing director	Leadership	3	< 0.001

The variables are categorized into three primary domains: Occupational and Organizational Factors, Health and Psychosocial Indicators, and Safety Culture and Leadership Dynamics. This structured approach ensures a comprehensive evaluation of the multifaceted determinants influencing head nurse decision fatigue.

#### 4.5.2. Regression Model Results

Multiple linear regression was performed using the enter method, with all 16 variables identified in Table 7 entered simultaneously as independent variables and TDF scores as the dependent variable. Collinearity diagnostics revealed tolerance levels ranging from 0.227 to 0.795 and VIFs from 1.258 to 4.412, confirming the absence of significant multicollinearity (all VIFs < 5).

The overall model demonstrated a good fit to the data (*R* ^2^ = 0.301, Adjusted *R* ^2^ = 0.238, *F*_16,127_ = 3.42, *p* < 0.001), indicating that the model accounted for approximately 30.1% of the variance in decision fatigue scores. Residual diagnostics confirmed the assumptions of linear regression: residuals were normally distributed (Shapiro–Wilk *W* = 0.986, *p* = 0.172), homoscedasticity was satisfied (Breusch–Pagan test, *p* = 0.213), and the Durbin–Watson statistic (1.942) indicated no significant autocorrelation of residuals.

As detailed in Table 8, the final regression model identified three variables significantly associated with decision fatigue. Among these, cultural heritage exhibited the strongest protective association (*β* = −0.296, *p* = 0.040). This was followed by physical status (inverse of fatigue symptoms) (*β* = −0.234, *p* = 0.019) and organizational and planning ability (*β* = −0.228, *p* = 0.009). The remaining 13 variables did not reach statistical significance (*p* > 0.05) in the multivariate model.

**TABLE 8:** Multiple linear regression analysis of factors associated with decision fatigue (*N* = 144).

Item	*B*	SE	*β*	*t*	*p*	95% CI
Constant	28.576	4.567		6.257	< 0.001	19.537–37.615
Physical status (absence of fatigue)	−1.085	0.458	−0.234	−2.369	0.019	−1.990–−0.179
Organizational and planning ability	−1.874	0.703	−0.228	−2.667	0.009	−3.264–−0.485
Cultural heritage	−0.343	0.165	−0.296	−2.078	0.040	−0.669–−0.016

Note on interpretation: The negative coefficients indicate that higher scores in these domains—representing stronger cultural capital, better physical condition (lower symptom frequency), and higher planning ability—are associated with reduced levels of decision fatigue. This constellation of factors forms the empirical basis for the “three‐tier protective framework.”

## 5. Discussion

### 5.1. Thresholds of Decision Load and the Triple Paradox of Resources: A Dual‐Pathway Model of Decision Fatigue

In the present cohort, the mean item score for decision fatigue was 0.99 ± 0.14, with a median of 9 (IQR = 7.25–10.75). This low‐to‐moderate level, characterized by minimal interindividual variance (CV = 14.1%), suggests a collectively adaptive load threshold. Such stability likely stems from structured clinical governance, such as the standardization of clinical pathways, which maintains decision‐making demands within homeostatic system boundaries.

Analysis of individual items revealed that “making decisions takes too much effort” (1.24) and “I can’t decide which option is best” (1.21) were the predominant symptomatic contributors. The former validates the core premise of ego depletion theory, wherein the exhaustion of finite cognitive resources precipitates a decline in self‐regulatory capacity [1]. The latter reflects decisional hesitancy and a pronounced loss aversion—a hallmark of healthcare management where the high stakes of patient safety compel nurse managers to elevate their internal decision thresholds. This phenomenon of “excessive caution,” often elusive in traditional rational‐choice models, represents an adaptive strategy forged at the intersection of medical safety culture and inherent cognitive constraints.

The practice environment was rated highly favorable (median = 225), with a narrow IQR (216–249) indicating strong evaluative consensus among head nurses. The high scores in “relationships with nurses” (5.42) underscore the efficacy of vertical team collaboration and the successful application of situational leadership in fostering stable support networks. Furthermore, the robust ratings for “organizational culture” (5.42) and “cultural heritage” (5.20) illustrate how healthcare institutions achieve the intergenerational transmission of cultural capital through ritualized practices, such as nursing rounds and multidisciplinary case discussions. This process aligns with Bourdieu’s field theory, specifically the mechanisms of habitus formation and field maintenance [18].

Conversely, the significantly lower scores for “adequate human and material resources” (4.30) and “fair workload” (4.84) expose a systemic triple paradox within the healthcare sector: the competing demands of fiscal austerity (constrained public hospital funding), quality imperatives (escalating safety standards), and human capital shortages (prolonged nursing professionalization cycles). Integrating these findings, we propose a dual‐pathway stress model as a theoretical framework for understanding decision fatigue in this population:

• Direct pathway: Workforce shortages ⟶ Task overload ⟶ Ego depletion ⟶ Decisional hesitancy (hypothesized).
• Indirect pathway: Perceived workload inequity ⟶ Heightened emotional labor ⟶ Cognitive interference ⟶ Decision energy depletion (hypothesized).

This model characterizes head nurses as “resource gatekeepers” caught within systemic constraints, suggesting that institutional interventions may be needed to disrupt this potentially deleterious feedback loop. However, as this is a cross‐sectional study, these pathways represent a theoretical interpretation rather than empirically established causal mechanisms.

### 5.2. Limited Explanatory Power of Demographic Variables: The Dominance of Professional Context and Occupational Assimilation

Traditional sociological theory, specifically the double burden hypothesis, posits that professional women experience compounded psychological strain arising from the intersection of workplace duties and domestic responsibilities, particularly child rearing [9]. However, our empirical findings diverge from this hypothesis. Despite the sample being predominantly female (95.14%), no statistically significant gender‐based differences in decision fatigue were observed (*p* = 0.745), nor did parental status demonstrate a measurable association (*p* = 0.180). This “dedifferentiation” suggests a process of occupational role homogenization, where the highly standardized nature of nursing management and institutional support mechanisms neutralize traditional gender and family role conflicts.

The head nurse role, characterized by high‐stakes responsibilities such as crisis intervention and complex resource coordination, appears to assimilate individual demographic differences into a unified professional identity. Regardless of gender, the uniformity of decisional demands—scheduling, staffing, and acute clinical management—creates a shared cognitive load. Furthermore, in East Asian cultural contexts, female managers may leverage compensatory familial support networks, such as intergenerational childcare provided by grandparents, which acts as a structural buffer. Additionally, the internalization of professional responsibilities may prevent domestic stressors from manifesting as quantifiable differences in decision fatigue within this specific cohort.

Similarly, decision fatigue scores remained invariant across age groups (*p* = 0.232) and educational levels (*p* = 0.840). These findings diverge from the conventional assumption that higher academic attainment necessarily expands cognitive reserves or enhances decision‐making efficiency. In the frontline clinical milieu, nursing leadership relies more heavily on tacit knowledge—such as interpersonal diplomacy and rapid situational judgment—than on formal academic theory. Consequently, a Master’s degree may not inherently provide a cognitive advantage in daily unit operations. Moreover, individuals with higher education levels often navigate a more diverse portfolio of research and teaching tasks; the intrinsic rewards and sense of professional accomplishment derived from these activities may offset the additional cognitive burdens of decision‐making.

Finally, the lack of association between blood type and decision fatigue (*p* = 0.925) provides empirical evidence against popular cultural claims, such as the “leadership advantage” hypothetically associated with Type O individuals. By finding no such link, our study aligns with the mainstream psychological consensus that blood type lacks empirical validity as a predictor of occupational behavior or personality‐driven stress responses [13].

### 5.3. From Structural Predicaments to Individual Empowerment: The Interplay of Institutional Hierarchy, Training, and Incentives

Hospital type emerged as a significant factor associated with decision fatigue (*p* = 0.013), with head nurses in regional tertiary hospitals (Grade 3‐B) exhibiting the highest scores (median = 10, IQR = 8.5–12.5). Positioned as the “middle layer” of China’s healthcare hierarchy, these institutions face a structural contradiction: they manage patient volumes comparable to elite tertiary care hospitals (Grade 3‐A) but operate with significantly lower fiscal subsidies, equipment levels, and staffing ratios. The convergence of policy‐driven pressures—such as hierarchical medical system pilot programs—and stringent resource constraints (e.g., cost controls based on diagnosis‐related groups [DRGs]) compels these managers to navigate high‐stakes decisions frequently with suboptimal resources. This “sandwich layer dilemma” suggests that the current polarized resource allocation strategy may inadvertently compromise the decision‐making quality of mid‐tier institutional leaders.

Individual empowerment through psychology‐related training was found to significantly mitigate decision fatigue (9 vs. 10, *p* = 0.009). Grounded in psychological capital (PsyCap) theory, such interventions bolster resources—including self‐efficacy, resilience, and hope—which serve as essential psychological buffers against occupational strain [19]. We posit that such training facilitates cognitive reappraisal, enabling head nurses to reframe decisional pressures as “challenges” rather than “threats,” thereby curtailing emotional exhaustion. Furthermore, enhanced emotional regulation skills conserve finite cognitive resources by reducing decisional anxiety, a phenomenon similarly observed in other high‐stress professional cohorts [20].

In contrast, management training yielded only a marginal effect (*p* = 0.050). While transformational leadership theory suggests that leadership development enhances managerial efficacy [21], the limited impact observed here may indicate a “skill‐demand mismatch.” Traditional training modules often emphasize process optimization (e.g., Plan‐Do‐Check‐Act (PDCA) cycles), whereas decision fatigue in head nurses frequently arises from unstructured problems, such as ethical dilemmas and complex interpersonal conflicts. Moreover, the internalization of management skills into heuristic decision habits requires prolonged longitudinal practice, which may result in a conversion lag undetectable in cross‐sectional designs.

The finding that income level did not correlate with decision fatigue (*p* = 0.316) challenges the doctrine of economic determinism. According to Self‐Determination Theory (SDT), external financial incentives often yield diminishing marginal returns for roles driven by high intrinsic motivation [22]. Decisional processes in nursing management are deeply anchored in professional altruism and the internalization of safety goals, making financial compensation insufficient to offset the cognitive and emotional “price” of leadership. Higher income often correlates with increased managerial complexity, creating a “high‐compensation, high‐load” equilibrium. Consequently, healthcare organizations should move beyond a reliance on monetary incentives, prioritizing non‐monetary support—such as enhanced decisional autonomy and formalized career development—to sustain the perceived meaningfulness of the head nurse role.

Finally, departmental specificity exerted no significant influence on fatigue levels (*p* = 0.703). While previous literature highlights the intensity of the ICU environment [23], we argue that ICU managers may develop specialized resilience mechanisms through chronic exposure to high‐acuity settings. Moreover, the trend toward institutional isomorphism—driven by national standards and Joint Commission International (JCI) accreditation—has led hospitals to adopt standard operating procedures (SOPs) that homogenize management practices across diverse units. This systemic convergence may diminish the impact of departmental differences on cognitive load, a hypothesis that warrants further longitudinal investigation.

### 5.4. The Spiral of Resource Depletion: Integrated Physiological–Psychological–Organizational Mechanisms

Head nurses reporting health issues exhibited a wider dispersion and higher levels of decision fatigue (*p* = 0.031). According to conservation of resources (COR) theory, chronic physiological stressors—such as insomnia or persistent pain—may continuously drain the reservoir of cognitive resources, potentially contributing to a “Loss Spiral” that erodes professional engagement and accelerates burnout [24]. However, causal interpretations should be made with caution given the cross‐sectional design. This is further evidenced by the significant escalation of decision fatigue scores alongside increased subjective fatigue (2 vs. 10, *p* = 0.003). Ego depletion theory posits that cognitive resources constitute a finite global pool; frequent, high‐stakes decision‐making leads to resource exhaustion, which ultimately manifests as decisional avoidance or compromised judgment quality [1, 25]. We propose a positive feedback loop—the “Fatigue–Fatigue Cycle”—wherein fatigue impairs decisional efficiency, thereby increasing residual workload and further exacerbating systemic exhaustion.

This cycle is underpinned by neuroendocrine mechanisms: chronic fatigue triggers prolonged activation of the hypothalamic–pituitary–adrenal (HPA) axis and the sympathetic nervous system. The resulting elevation in allostatic load (e.g., chronic cortisol exposure) inhibits prefrontal cortex functions—specifically working memory and executive control—while simultaneously sensitizing amygdala‐driven emotional reactivity [26, 27]. Consequently, decision fatigue is significantly more pronounced in those with diminished job satisfaction (*p* < 0.001) and suboptimal psychological states (*p* = 0.004). Low satisfaction reflects a perceived scarcity of extrinsic rewards (e.g., recognition and career progression), which weakens the individual’s capacity to mediate high‐arousal demands, such as physician–patient conflicts.

Interestingly, our findings diverge from previous studies [12] as exercise frequency and sleep duration did not significantly impact decision fatigue (*p* > 0.05). This suggests an “overshadowing effect” where the extreme cognitive load inherent in nursing management may negate the marginal restorative benefits of baseline exercise or sleep quantity. Furthermore, it implies that the “quality” of recovery—such as high‐intensity interval training (HIIT) or deep‐sleep efficiency—may be more critical than duration in alleviating cognitive congestion.

Notably, the high decision fatigue observed among those reporting “low stress” (*p* = 0.002) challenges the linear stress‐fatigue hypothesis. This paradox necessitates a typological shift in stress research: distinguishing between chronic adaptive stress (which may lead to emotional numbing) and acute high‐arousal stress (which causes cognitive shattering). Future research should transcend the limitations of subjective psychometric scales by incorporating multimodal physiological data (e.g., heart rate variability and cortisol levels) to capture the nuanced interaction between physiological depletion and psychological fatigue.

### 5.5. A Proposed Three‐Tier Protective Framework: An Integrated Interpretation

Multiple linear regression analysis identified three variables significantly associated with lower decision fatigue scores. Cultural heritage (*β* = −0.296) showed the strongest negative association, followed by physical resilience (absence of fatigue symptoms) (*β* = −0.234) and organizational planning ability (*β* = −0.228). These associations suggest a potentially synergistic, multilevel protective framework—integrating organizational–cultural capital, cognitive–managerial metaskills, and individual physiological–emotional resources—that may buffer against decisional exhaustion. However, as these findings are derived from cross‐sectional data, they should be interpreted as associations rather than causal effects.

#### 5.5.1. Potential Clinical Implications and Strategic Considerations

The identification of these associated factors provides a structured framework for considering targeted institutional interventions. While causal efficacy remains to be established in future research, the following strategies may be worth exploring.

##### 5.5.1.1. Tier 1: Fortifying the Organizational‐Cultural Foundation

Given the potent protective association of cultural heritage, hospital leadership must move beyond superficial branding to proactively cultivate a culture that tangibly values nurse managers. This can be achieved by institutionalizing mentorship programs that facilitate the intergenerational transmission of resilience and establishing “psychological safe‐havens”—regular forums where leaders can collectively navigate complex problem‐solving without fear of administrative reprisal.

##### 5.5.1.2. Tier 2: Enhancing Cognitive–Managerial Metaskills

Training curricula must evolve from routine administrative procedures to the development of higher‐order metacognitive skills, specifically organizational planning. Emphasis should be placed on “cognitive triage”—the ability to dynamically prioritize under high‐pressure conditions—and utilizing high‐fidelity simulation to navigate unstructured clinical–ethical dilemmas.

##### 5.5.1.3. Tier 3: Bolstering Psychosomatic and Emotional Resources

Support at the individual level must be systemic rather than incidental. Organizations should consider incorporating psychological resilience profiles into succession planning and selection processes for head nurses, specifically identifying traits such as emotional stability (defined as the capacity to maintain emotional balance and functionality under stress). Evidence‐based psychological training (e.g., mindfulness‐based stress reduction or cognitive‐behavioral coaching) should be mandated as a core competency. Critically, structural policies must address the root causes of physical depletion by ensuring equitable workloads and access to wellness programs, thereby preserving the physiological reserves necessary for high‐quality decision‐making.

#### 5.5.2. Future Research Directions

Our findings, particularly the dual‐pathway model and the three‐tier protective framework, chart several trajectories for subsequent inquiry.

##### 5.5.2.1. Interventional Efficacy

Future research should test whether theory‐driven interventions targeting these hypothesized pathways are effective in reducing decision fatigue. For the direct pathway (staffing shortages ⟶ task overload ⟶ fatigue), studies could examine the impact of AI‐driven decision‐support systems or protected administrative time. For the indirect pathway (perceived inequity ⟶ emotional labor ⟶ fatigue), the efficacy of structured team debriefings warrants investigation.

##### 5.5.2.2. Longitudinal and Mixed‐Methods Designs

Longitudinal studies are essential to establish causality and track the evolution of decision fatigue across different career stages. Complementary qualitative inquiries are needed to capture the nuanced lived experiences of nurse managers within these complex systemic pathways.

##### 5.5.2.3. Construct Refinement

Subsequent work should utilize more granular instruments (e.g., PsyCap scales) to dissect which specific facets of “emotional stability” or “planning ability” are most prophylactic against fatigue.

### 5.6. Limitations

Despite the significant insights provided by this study, several limitations warrant consideration. Primarily, the cross‐sectional design precludes the establishment of definitive causal inferences. Because decision fatigue is a dynamic phenomenon, this methodology cannot capture its temporal evolution or longitudinal trajectories across different career stages. To address this, future research must employ longitudinal and prospective designs to elucidate causal mechanisms and the long‐term stability of the identified predictors. Furthermore, our reliance on self‐reported data introduces the potential for response bias. Participants’ subjective evaluations of their fatigue, health status, and psychological well‐being may be influenced by social desirability or recall inaccuracies. Subsequent studies would significantly benefit from complementing psychometric scales with objective physiological indicators (e.g., heart rate variability and salivary cortisol) to provide a more multidimensional assessment of decision fatigue and its underlying mechanisms.

Another critical set of constraints relates to the sample composition and size. The cohort was predominantly drawn from a specific geographical region in China, which necessitates caution when extrapolating these findings to broader healthcare systems or diverse cultural contexts. Within this sample, the pronounced gender imbalance (95.14% female) potentially obscures gender‐specific dynamics relevant to male head nurses. Beyond these overarching demographic trends, the relatively small sample sizes in certain clinical subgroups—particularly among the operating and recovery room staff (*n* = 3)—attenuate the statistical power required to detect specific effects and limit the precision of our subgroup estimates. This localized constraint is reflected in the inability to calculate reliable IQRs for these specific cohorts. To resolve these demographic and structural limitations, future multicenter inquiries must strive for larger, geographically diverse, and gender‐balanced cohorts with robust representation across all departmental categories to enable more rigorous subgroup analyses.

## 6. Conclusions

This study suggests that decision fatigue among head nurses may be understood through a proposed dual‐pathway framework: a direct pathway involving task overload and ego depletion and an indirect pathway involving emotional labor and cognitive interference. Notably, psychological training was significantly associated with lower fatigue levels, potentially through the bolstering of PsyCap.

Central to our findings is the identification of three factors associated with lower decision fatigue: organizational–cultural capital, cognitive–managerial metaskills, and individual psychosomatic resources. These associations suggest a potentially synergistic protective framework, though causal relationships should be examined in future longitudinal studies. To effectively mitigate decision fatigue, a multilevel strategic approach is imperative. Healthcare administrators should prioritize the cultivation of a genuinely supportive organizational culture, integrate advanced psychological and metacognitive training into leadership development curricula, and incorporate emotional resilience as a core criterion in nursing leadership selection and succession planning. Future research should prioritize the development and rigorous evaluation of targeted interventions tailored to the specific dynamics of the dual‐pathway model.

## 7. Implications for Nursing Management

Based on the associations identified in this study, we propose a multilevel practical framework to mitigate decision fatigue among nurse managers. At the individual level, leaders may employ “cognitive triage” to conserve mental bandwidth by standardizing routine administrative tasks through algorithmic outsourcing and SOPs, alongside interrupting the fatigue cycle via brief intrashift microrestoration pauses and cognitive reappraisal. At the institutional level, administrators should strengthen organizational culture—the strongest identified protective factor—by establishing structured mentorships and “psychological safe havens” for peer debriefing. Furthermore, institutions must evolve leadership curricula to include metacognitive and evidence‐based psychological skills, while addressing structural constraints through optimized staffing models and nonmonetary recognition. Finally, at the policy level, healthcare systems should re‐examine resource allocation strategies to alleviate the disproportionate structural burdens placed on midtier (Grade 3‐B) hospital leaders, alongside driving systemic investments in wellness programs and longitudinal research to safeguard the long‐term decision‐making capacity of frontline clinical managers.

## Author Contributions

Wenyu Li: conceptualization, methodology, investigation, writing–original draft, writing–review and editing, and supervision. Menfang Li: conceptualization, methodology, investigation, writing–original draft, and writing–review and editing. Jingjing Ma: investigation, data curation, and writing–review and editing. Xiaoyu Zhou: investigation, data curation, and writing–review and editing. Yani Yan: investigation, data curation, and writing–review and editing. Shaozhi Tian: investigation, data curation, and writing–review and editing. Kemiao Dai: investigation, data curation, and writing–review and editing. Zhiyang Xie: investigation, data curation, and writing–review and editing. Huirong Xie: investigation, data curation, and writing–review and editing. Chunqing Yang: investigation, data curation, and writing–review and editing. Longcha Liu: investigation, data curation, and writing–review and editing. Zhengjie Dai: investigation, data curation, and writing–review and editing. Sula Li: investigation, data curation, and writing–review and editing. Xiaoqun Xu: conceptualization, methodology, writing–review and editing, supervision, and project administration.

## Funding

This research did not receive any specific grant from funding agencies in the public, commercial, or not‐for‐profit sectors.

## Consent

The authors have nothing to report.

## Conflicts of Interest

The authors declare no conflicts of interest.

## Data Availability

The datasets used and/or analyzed during the current study are available from the corresponding authors upon reasonable request.
